# Quantitive disease resistance (QDR): The alternative to “all-or-nothing” strategy in plant immunity

**DOI:** 10.1093/plcell/koaf132

**Published:** 2025-05-21

**Authors:** Pei Qin Ng

**Affiliations:** Assistant Features Editor, The Plant Cell, American Society of Plant Biologists; Department of Plant Sciences, University of Cambridge, Cambridge CB2 3EA, UK

Complete resistance against plant diseases is often mediated by 1 or several key genes, such as NOD-like receptors (NLRs), a major class of race-specific quantitative resistance genes (R genes) (Veneault-Fourrey and Rep 2021; Michel et al. 2023). However, there is an alternative to this “all-or-nothing” disease resistance in plants, in which plants respond at a varying degree to an infection. Some plants will show a better resistance response compared to others but will not completely avoid disease symptoms (Gou et al. 2023; Michel et al. 2023). This range of immune response is achieved through the cumulative, minute effects of many genes and is known as quantitative disease resistance (QDR) (Corwin and Kliebenstein 2017; Gou et al. 2023). However, our understanding of the underlying molecular mechanisms and transcriptome landscape remains limited.

To address this knowledge gap in understanding QDR, **Florent Delplace and colleagues** (Delplace et al. 2025) generated and analyzed transcriptome-wide changes of 23 Arabidopsis (*A. thaliana*) accessions infected with the necrotrophic fungus *Sclerotinia sclerotiorum*. The 23 accessions showed variability in the number of differentially expressed genes (DEGs), ranging from approximately 6,500 to 9,200, with genes predominantly downregulated upon *S. sclerotiorum* inoculation. Interestingly, phylogenetic comparisons of the transcriptome signature of the 23 accessions did not show consensus transcriptomic changes, suggesting considerable expression diversity across the different accessions. Nevertheless, all of the accessions consistently showed infection-induced transcriptome reprogramming, albeit at different levels of susceptibility to the *S. sclerotiorum*. The authors then looked for conserved gene expression changes across the 23 accessions and identified 1,957 DEGs, referred to as the core DEGs, that were consistently differentially expressed in all accessions. The core DEGs consist of 1,049 upregulated genes and 908 downregulated genes. Gene Ontology (GO) analysis of the upregulated core DEGs revealed that these DEGs were involved in primary and secondary metabolism, transport, and defense responses (Figure 1A). The authors highlighted that these processes are conserved across species and contribute to broad-spectrum defense against fungal infection in their discussion. Contrastingly, the downregulated core DEGs include those associated with responses to light, developmental processes, and primary photosynthetic metabolism (Figure 1B).

**Figure.**
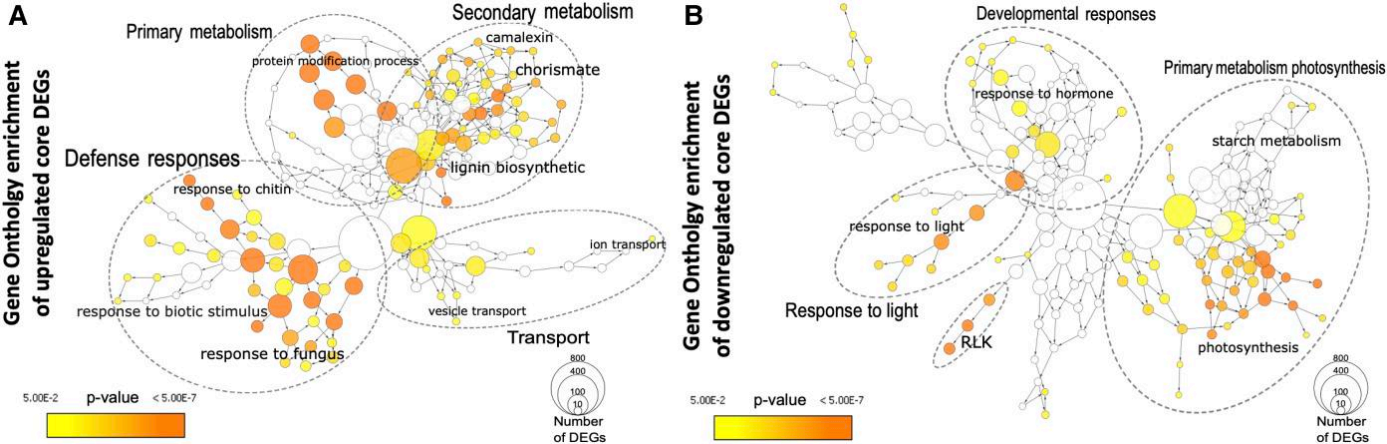
Gene ontology (GO) results for upregulated **(A)** and downregulated **(B)** core DEGs. Adapted from Delplace et al. (2025), Figure 3.

To further refine the accession transcriptome changes in response to the infection, they performed a weighted correlation network analysis to identify transcriptome-wide coexpression gene modules. A total of 48 coexpression gene modules were identified. However, the gene expression changes associated with these modules varied across different accessions. They identified 4 major modules corresponding to the GO terms response to stress (module 1), hormone signaling and primary metabolism (module 2), development and gene expression regulation (module 3), and immune responses (module 4). The authors further investigated cis-regulation in QDR by analyzing the DNA affinity purification cis-motif from the promoters of genes identified in each module. No strong association with a common motif was identified, but rather several motifs across different modules, further highlighting the diversity in QDR responses among accessions. This analysis shows that the conserved QDR comprises of a mix of different pathways.

In summary, the work of Delplace et al. (2025) revealed intricate transcriptome changes across 23 Arabidopsis accessions and identified a core set of genes mediating QDR in plants. In the discussion of their work, the authors reflect on how their results help shed light on some outstanding questions about QDR. This work will be informative in paving future research into understanding the complex mechanism of QDR in plants and how we can use it to inform crop breeding strategies.

## Recent related articles in *The Plant Cell*

- Wang et al. (2024) provided insight into how an intracellular NLR-immune receptor forms a complex with other immunity proteins, then acts as a transcription factor NbWRKY40e to mediate stomatal immunity in *Nicotiana benthamiana*
- Sun et al. (2024) showed the effect of alternative splicing in potato NLR gene *RB* in plant immunity.
- A review by Dodds et al. (2024) explained the principles of plant immunity and key research milestones in the field to date.

## Data Availability

No new data were generated or analysed in support of this article.
